# Retraction: Rtt107 Phosphorylation Promotes Localisation to DNA Double-Stranded Breaks (DSBs) and Recombinational Repair between Sister Chromatids

**DOI:** 10.1371/journal.pone.0176035

**Published:** 2017-04-12

**Authors:** Pranav Ullal, Felipe Vilella-Mitjana, Adam Jarmuz, Luis Aragón, PLOS ONE Editors

Concerns have been raised about the integrity of images presented in Figures 2 and S1 of this article. The original blot images from which the figures were made are not available and the first author has indicated that the images in the figures are not from the samples described in the article. At the corresponding author's request, the institutions involved are investigating this matter. The corresponding author and representatives from the institution have advised that the article should be retracted due to the concerns identified about the two figures and the reliability of the underlying data.

In light of the concerns raised, the authors and the *PLOS ONE* Editors retract this article.
